# Dopamine Transporter Imaging Predicts Mortality in a Long-Term Follow-Up after Subthalamic Deep Brain Stimulation in Parkinson’s Disease

**DOI:** 10.1159/sfn/aeuag002

**Published:** 2026-08-05

**Authors:** Birgitte Liang Chen Thomsen, Steen Rusborg Jensen, Anders Lundetoft Clausen, Sine Fjeldhøj, Bo Jespersen, Merete Karlsborg, Markus Nowak Lonsdale, Annemette Løkkegaard

**Affiliations:** a Department of Neurology, Copenhagen University Hospital, Bispebjerg and Frederiksberg, Copenhagen, Denmark; b Department of Neurosurgery, Copenhagen University Hospital, Copenhagen, Denmark; c Department of Clinical Physiology and Nuclear Medicine, Copenhagen University Hospital, Bispebjerg and Frederiksberg, Copenhagen, Denmark; d Department of Clinical Medicine, Copenhagen University, Copenhagen, Denmark

**Keywords:** Parkinson’s disease, Deep brain stimulation, Dopamine transporter imaging, Long-term follow-up

## Abstract

**Introduction:**

Deep brain stimulation of the subthalamic nucleus (STN-DBS) is an efficient treatment for advanced Parkinson’s disease (PD), when oral medication no longer provides satisfactory symptom control or causes disabling side effects. However, long-term outcomes vary considerably, and reliable preoperative biomarkers remain lacking. Dopamine transporter single-photon emission computed tomography (DAT SPECT) provides an indirect in vivo measure of presynaptic striatal dopaminergic degeneration in the basal ganglia. In patients evaluated for STN-DBS, DAT SPECT with [^123^I]FP-CIT supported the clinical diagnosis. This study aimed to examine whether preoperative DAT SPECT predicts the long-term outcome after STN-DBS surgery.

**Methods:**

Fifty-five patients with PD underwent DAT SPECT before STN-DBS surgery. Motor symptoms were assessed in ON and OFF medication states before surgery, and at long-term follow-up, 8–14 years after surgery. Cognition was assessed before surgery and at the long-term follow-up, and clinical data were curated retrospectively from medical records.

**Results:**

Lower DAT striatal specific binding ratio (SBR) significantly correlated with longer disease duration, indicating greater dopaminergic loss. Reduced striatal SBR predicted increased mortality during the long-term follow-up. Regional analyses revealed that this association was driven by the caudate nucleus. Furthermore, greater caudate compared to putaminal involvement, reflected by a higher putaminal/caudate uptake ratio, was related to longer disease duration and increased mortality. Preoperative DAT SPECT measures were not associated with DBS effect, cognitive function, or depression.

**Conclusion:**

Dopamine transporter imaging provides valuable insight into the pathophysiology and disease progression of PD. Although DAT SPECT did not predict the therapeutic effect of STN-DBS, this study demonstrates that DAT SPECT captures aspects of disease progression and survival in PD. These results highlight the value of assessing the dopaminergic system before advanced treatment, as DAT SPECT may provide prognostic indicators that could complement the preoperative evaluation.

## Plain Language Summary

Parkinson’s disease (PD) is a brain disorder that causes movement problems such as shaking and slowness of movement due to degeneration of dopamine cells. In the later stages of the disease, medication works less effectively or causes side effects. At that point, some people may benefit from a surgical treatment called deep brain stimulation (DBS). This procedure involves placing small electrodes in a part of the brain called the subthalamic nucleus to help control symptoms. Before surgery, patients usually have a brain scan called dopamine transporter single-photon emission computed tomography (DAT SPECT) to confirm the diagnosis. This scan shows dopamine transporters, which indicate how much dopamine cell loss has occurred in the brain. This study investigated whether DAT SPECT taken before DBS surgery could predict the long-term outcome. Fifty-five people with PD had a DAT SPECT scan before surgery. Their movement and cognitive abilities were tested before and again 8–14 years after surgery. The scans showed that patients with a longer disease duration had greater loss of dopamine cells. Those with more damage in one brain region, the caudate nucleus, had a higher risk of death. However, the scans did not predict who would benefit most from DBS or who might experience dementia or depression over time. Overall, the study shows that DAT SPECT provides useful information about brain changes in PD and can predict mortality after DBS surgery; however, it does not predict the treatment effect.

## Introduction

Parkinson’s disease (PD) is a neurodegenerative disease with a clinical diagnosis based on the cardinal motor symptoms: bradykinesia, rigidity, and tremor [1]. These motor symptoms are primarily related to changes in the dopaminergic system in the basal ganglia [2]. In dopamine transporter single-photon emission computed tomography (DAT SPECT), [^123^I]FP-CIT is used to measure presynaptic dopamine transporter loss in the striatum, comprising the putamen and caudate nucleus. Thus, DAT SPECT is used to support the diagnosis of PD [3], and a normal DAT SPECT precludes the diagnosis [4].

In early stages of PD, dopaminergic medication is usually effective on motor symptoms; however, after 5–10 years of treatment, the majority of patients develop fluctuations and dyskinesia [5]. Stereotactic neurosurgery with implantation of deep brain stimulation electrodes in the subthalamic nucleus (STN-DBS) is a safe and effective treatment for advanced PD. STN-DBS treatment improves dyskinesia and fluctuations by reducing the need for medication [6]. Randomized controlled studies have demonstrated that STN-DBS is more effective than best medical treatment in alleviating motor symptoms and improving quality of life [7, 8]. Furthermore, long-term follow-up studies have demonstrated the sustained efficacy of STN-DBS for up to 15 years, including in the late stages of PD [9–15].

We recently published long-term follow-up data on STN-DBS clinical outcomes more than 8 years after surgery in a cohort of patients with PD [9, 15]. At the preclinical examination, DAT SPECT was acquired to confirm the diagnosis of PD. The aim of this study was to examine whether preoperative DAT SPECT is related to the long-term outcome of STN-DBS and predictive of subsequent disease progression. We hypothesized that lower preoperative striatal dopamine transporter binding would be associated with worse long-term clinical outcomes after STN-DBS, including greater cognitive decline and higher mortality. To our knowledge, this is the first long-term follow-up study of DBS to report neuroimaging, and the analysis of whether preoperative DAT SPECT can predict the treatment effect of STN-DBS is exploratory.

## Methods

### Participants and Inclusion Criteria

Fifty-five patients at Bispebjerg University Hospital were referred to STN-DBS surgery between 2002 and 2008. Patients fulfilled the inclusion criteria for STN-DBS surgery based on the original recommendations from the Core Assessment Program for Surgical Interventional Therapies (CAPSIT) protocol [16]: A diagnosis of levodopa-responsive PD for at least 5 years, motor fluctuations and levodopa-induced dyskinesia, Hoehn and Yahr stage ≥3.5, and no dementia (Mini-Mental State Examination [MMSE] >24). Exclusion criteria were as follows: significant psychiatric disorders such as treatment-resistant depression and contraindications to surgery. Motor symptoms of PD were objectively rated using the Unified Parkinson’s Disease Rating Scale (UPDRS) part III (UPDRS motor score). The UPDRS assessment was performed with medication in a defined “best ON state” (Med ON), which was the time of peak effect after intake of the patient’s normal morning dose of antiparkinsonian medication and in a defined OFF state (Med OFF) after 12 h of discontinuation of medication [15]. Furthermore, neuropsychological testing, including the Mattis Dementia Rating Scale (DRS), was performed in the Med ON state to exclude dementia. Patients underwent DAT SPECT (mean ± SD: 8 ± 11 months) before surgery as part of the clinical workup (shown in Table 1).

**Table 1.** Patient characteristics

Variable	Mean±SD (range) or *n* (%)
Female/male ratio	20/35 (36/64%)
Age at surgery, years	60.5±7.14 (43.7–75.1)
Age at PD onset, years	47.0±7.48 (30–63)
Disease duration at surgery, years	13.1±4.70 (6–24)
Preoperative LEDD, mg	1,025±460 (333–2,197)
Preoperative Med OFF motor UPDRS	42.9±10.8 (17–67)
Preoperative most affected striatal SBR	1.22±0.35 (0.56–2.22)
Preoperative most affected putaminal/caudate uptake ratio	0.76±0.12 (0.5–1.05)
Alive/dead at long-term follow-up	30/25 (55/45%)
Age at death, years	71.5±5.25 (62.0–82.7)

Demographics and clinical characteristics of the study population. Preoperative striatal SBR and putamen/caudate uptake ratio are reported from the hemisphere with the lowest striatal SBR.

LEDD, levodopa equivalent dose; Med OFF, Off medication; motor UPDRS, motor part of Unified Parkinson’s Disease Rating Scale; SBR, striatal binding ratio.

### Surgical Procedure

The surgical procedure was described in detail by Thomsen et al. [15]. Surgery proceeded under local anesthesia after 12 h of discontinuation of antiparkinsonian medication. Quadripolar DBS electrodes were implanted bilaterally using magnetic resonance imaging-guided stereotactic surgery. The electrode location was optimized using microelectrode recording, clinical testing, and X-ray. In the first 35 patients who underwent STN-DBS surgery at our center, DAT SPECT was repeated 1 year after surgery. The short-term follow-up data from this subgroup were presented in 2007 [17].

### Clinical and Neuropsychological Assessment

A clinical long-term follow-up was performed 8.5–13.6 years (mean ± SD: 11.0 ± 1.6) post-surgery in 24 patients. At the time of the long-term follow-up, UPDRS in Med OFF-DBS OFF, Med OFF-DBS ON, and Med ON-DBS ON were performed. The long-term follow-up was performed similarly to the 1-year follow-up; however, DBS stimulation was only turned off for as long as the patient could tolerate and no longer than 3 h. Furthermore, neuropsychological examinations were performed in the Med ON-DBS ON state. Thirty STN-DBS operated patients were still alive, and 25 had died before the long-term follow-up was conducted. Medical records were retrospectively reviewed until the time of death or until July 1, 2017. The mean time of observation was 12.0 ± 1.7 years. Medication, time of death, cause of death, and cognitive status were extracted. Treatment effects were calculated as the difference between the UPDRS motor score with and without medication/stimulation. Antiparkinsonian medications were converted into the levodopa equivalent daily dose [18]. Depression and dementia were defined by initiation of medical treatment or a diagnosis made by the neurologist based on clinical rating scales and medical history. The DAT SPECT imaging data were compared with the curated clinical data [15].

### DAT SPECT Acquisition and Analysis

Image acquisition and analysis were performed as previously described [17]. A 30-min SPECT acquisition with concurrent attenuation correction was performed 3 h after thyroid blockade and intravenous injection of [^123^I]FP-CIT. The reconstructed 3D volume was aligned along the orbitomeatal line. The resulting data were cropped to 1 slice covering 12.5 mm of the basal ganglia in the axial direction and analyzed semiquantitatively using a region of interest (ROI) method (shown in Fig. 1). The specific binding ratios (SBRs) of [^123^I]FP-CIT uptake in the striatum, putamen, and caudate nucleus were calculated as the ratio between the uptake and the nonspecific background uptake, reduced by one. The ROI positioning was performed by the same experienced technician in all examinations. The background uptake was estimated from the median pixel value of brain tissue in the analyzed slice. Furthermore, the uptake ratio between putamen and caudate nucleus was calculated (putaminal/caudate uptake ratio). Only the hemisphere with the lowest uptake was used for analysis.

**Fig. 1.**
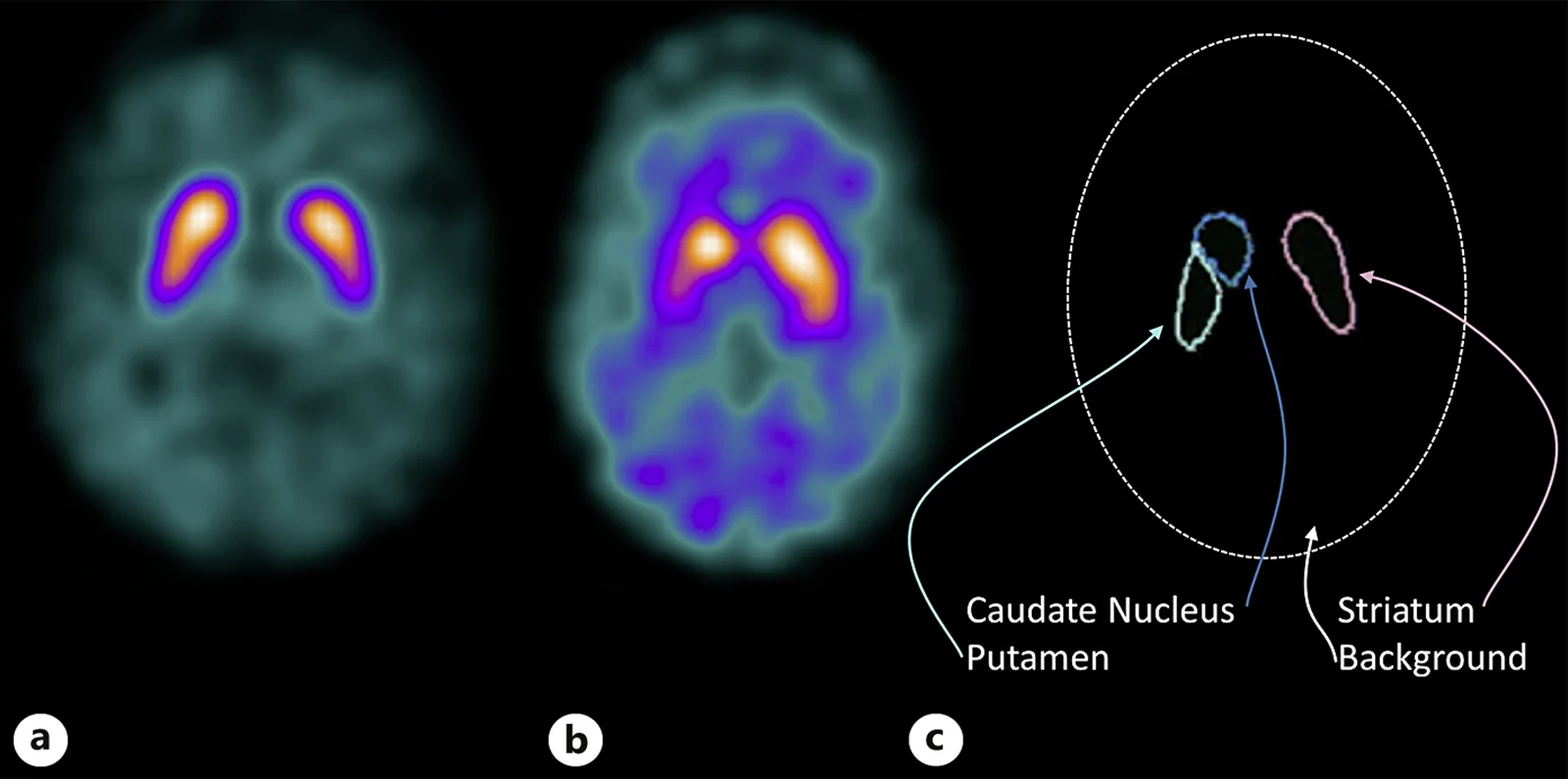
Dopamine transporter single-photon emission computed tomography. Examples of dopamine transporter single-photon emission computed tomography (DAT SPECT) acquired 3 h after injection of 185MBq [^123^I]-FP-CIT. **a** Normal DAT SPECT. **b** Patient with PD showing bilateral reduction of striatal uptake most pronounced in the right hemisphere. **c** Regions of interest (ROIs) used for analysis.

### Statistical Analyses

Associations between DAT SPECT measures (SBR and putaminal/caudate uptake ratio) and clinical variables (disease duration, UPDRS motor score, treatment effect, Mattis DRS score, and depression) were analyzed using general linear models. All models included sex, preoperative Med OFF or ON UPDRS motor score, and age, disease duration, and LEDD at the time of the DAT SPECT as covariates unless otherwise specified. As intake of serotonergic drugs has been shown to increase the striatal-to-nonspecific binding ratio of [^123^I]FP-CIT binding by approximately 10% [19, 20], the general linear model of depression was also performed using a selective serotonin reuptake inhibitor (SSRI)/serotonin-norepinephrine reuptake inhibitor (SNRI)-corrected SBR (SBR divided by 1.1). Mortality was analyzed using a multivariate Cox regression model. Striatum, putamen, and caudate SBRs were log-transformed using base 1.1 to improve linearity, and the hazard ratios are presented per 10% relative increase. The hazard ratio of the putamen/caudate uptake ratio is presented per 0.1 absolute increase. Although log transformation improved linearity for the SBRs, neither variable achieved perfect linearity, potentially reflecting the relatively small number of participants in this cohort, and the results should be interpreted with some caution. Dementia was analyzed using Cox regression competing-risk analysis with death as the competing event. For both survival analyses of dementia and mortality, the time from the preoperative DAT SPECT was used as the time variable. Although dopaminergic degeneration in PD is likely exponential rather than linear [21], it can be assumed to approximate linearity in later stages of the disease.

Statistical analyses were performed using R (R Foundation, 3.6.1, Vienna, Austria) and RStudio (RStudio PBC, 1.1.383, Boston, MA, USA). Results are presented as mean ± standard deviation (SD), and ranges in parentheses unless otherwise specified. A *p* value <0.05 was considered statistically significant.

## Results

### Baseline DAT SPECT Findings

The mean striatal SBR of the most affected side before surgery was 1.22 ± 0.35, and the putaminal/caudate uptake ratio was 0.76 ± 0.12. This corresponds to a 34% reduction compared to healthy controls. In a healthy population, the putaminal/caudate uptake ratio has a mean of 1.00 ± 0.05 (range 0.90–1.06; previously unpublished data from a population of healthy controls), whereas in PD, it decreases to <1.0 at the time of diagnosis [22].

For all analyses, models included sex, age, disease duration, preoperative Med OFF UPDRS motor score, and LEDD as covariates, as described in the Methods section. The only exception was the model including the Med ON UPDRS motor score, which did not include the Med OFF UPDRS motor score. The preoperative striatal SBR and putaminal/caudate uptake ratio were significantly correlated with the disease duration at the time of the DAT SPECT, after adjustment for covariates. Lower striatal SBR was associated with longer disease duration (*p* = 0.02, β = −0.024 [95% CI: −0.026 to −0.022]) (shown in Fig. 2a). A higher putaminal/caudate uptake ratio was associated with a longer disease duration (*p* = 0.029, β = 0.0072 [95% CI: 0.00095–0.014]) (shown in Fig. 2b). Lower caudate SBR was also associated with longer disease duration (*p* = 0.017, β = −0.037 [95% CI: −0.071 to −0.0036]), whereas lower putaminal SBR was not (*p* = 0.14). When adjusting for sex, age, disease duration, and LEDD, no correlations were found between the preoperative striatal SBR and the Med OFF (*p* = 0.91) or Med ON UPDRS motor scores (*p* = 0.28).

**Fig. 2.**
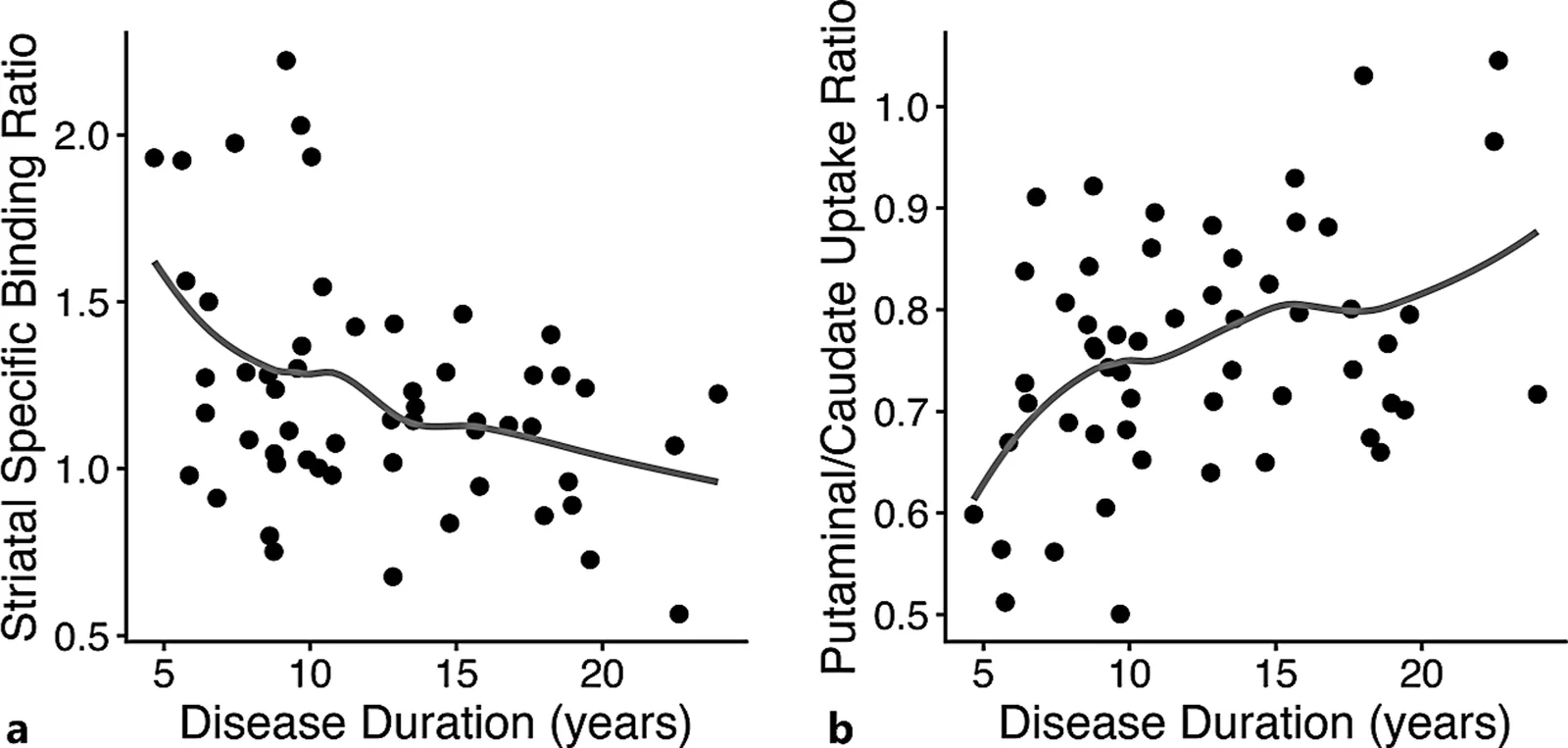
DAT SPECT correlates to duration of disease. Descriptive data of the dopamine transporter (DAT) SPECT corresponding to disease duration at preoperative DAT SPECT. **a** Scatterplot of striatal specific binding ratio (SBR) and the disease duration with a smooth curve. **b** Scatterplot of putaminal/caudate uptake ratio and the disease duration with a smooth curve. Each dot denotes 1 patient.

### Treatment Effects

Adjusted for sex, age, disease duration, preoperative Med OFF UPDRS motor score, and LEDD, no significant correlations were found between preoperative striatal SBR and medication effect (*p* = 0.35), nor between the preoperative striatal SBR and the DBS effect 1 year (*p* = 0.46) or 10 years after surgery (*p* = 0.95).

### Cognitive Outcomes

Thirty out of 55 patients (55%) had developed a clinical diagnosis of dementia before the long-term follow-up or before the time of death. The mean time from surgery to the diagnosis of dementia was 4.6 ± 2.7 years. When adjusting for sex, age, disease duration, preoperative Med OFF UPDRS motor score, and LEDD, no significant correlation was found between the preoperative striatal SBR and dementia (*p* = 0.46), putaminal/caudate uptake ratio and dementia (*p* = 0.36), or caudate SBR and dementia (*p* = 0.75).

Fifty-two patients had the Mattis DRS performed before the preoperative DAT SPECT. No significant correlation was found between the preoperative Mattis DRS and preoperative striatal SBR (*p* = 0.29), putaminal/caudate uptake ratio (*p* = 0.90), or caudate SBR (*p* = 0.29). Twenty-three patients had the Mattis DRS performed at the long-term follow-up. In these patients, no significant correlation was found between the long-term Mattis DRS and preoperative striatal SBR (*p* = 0.20), putaminal/caudate uptake ratio (*p* = 0.44), or caudate SBR (*p* = 0.29) adjusted for covariates as above.

### Depression

Seventeen patients were diagnosed with depression before surgery. Adjusted for sex, age, disease duration, preoperative Med OFF UPDRS motor score, and LEDD, no significant differences were found for striatal SBR (*p* = 0.11) and putaminal/caudate uptake ratio (*p* = 0.094) between patients with or without depression. All patients with depression received antidepressants. Correcting for the use of SSRI/SNRI did not change the result.

### Mortality

Twenty-five patients had died before the long-term follow-up was conducted. Cause of death was attributed to pneumonia (*n* = 9), old age (*n* = 4), cancer (*n* = 5), stroke (*n* = 1), complications during PEG placement (*n* = 1), suicide (*n* = 1), pulmonary embolism (*n* = 1), or was of unknown cause (*n* = 3).

Multivariate Cox regression analyses were performed for preoperative striatal, putamen and caudate SBR, and putamen/caudate ratio, each corrected for sex, age, disease duration, preoperative Med OFF UPDRS motor score, and LEDD. Higher striatal DAT binding showed a protective association with reduced mortality, with each 10% increase in striatal SBR corresponding to a 17% decreased risk of death (hazard ratio [HR] = 0.83, 95% CI: 0.70–0.98, *p* = 0.027). The model demonstrated good discriminative ability (C-index: 0.74, SE: 0.05).

When putamen and caudate were analyzed separately, putamen SBR was not significantly associated with mortality (*p* = 0.15). In contrast, caudate SBR emerged as a significant predictor, with each 10% increase in caudate SBR corresponding to a 16% decreased risk of death (HR = 0.84, 95% CI: 0.73–0.97, *p* = 0.019). This model showed similarly good discrimination (C-index: 0.73, SE: 0.05).

The pattern of dopaminergic denervation, reflected by the putaminal/caudate ratio uptake ratio, was significantly associated with higher mortality. A higher putaminal/caudate uptake ratio, reflecting a relative reduction in caudate uptake compared with putaminal uptake, was associated with increased mortality risk, with each 0.1 unit increase corresponding to a 58% increased risk of death (HR = 1.58, 95% CI: 1.02–2.44, *p* = 0.04). The model demonstrated good discriminative ability (C-index: 0.73, SE: 0.05).

## Discussion

We have conducted a long-term follow-up study of patients with PD who underwent STN-DBS, with a mean follow-up time of 11 years. We showed that preoperative dopamine transporter imaging was associated with mortality but not the long-term treatment effect of STN-DBS. Preoperative dopamine transporter uptake significantly correlated with disease duration, such that longer disease duration corresponded to a lower striatal uptake, in accordance with previous studies [3, 23].

In the healthy population, the putaminal/caudate binding ratio is approximately 1. With the onset of PD, dopaminergic denervation begins primarily in the putamen [24]. The initial sparing of the caudate nucleus results in a reduced putaminal/caudate uptake ratio, as reflected by the preoperative mean value of 0.76 in the present cohort. With disease progression, this ratio changes over time. Our data, from patients with PD with disease durations ranging from 6 to 24 years, indicate that with longer disease duration, the ratio may again approach 1 (illustrated in Fig. 2b). These findings suggest that the progression of the putaminal/caudate uptake ratio may follow a U-shaped rather than linear pattern. A hypothetical longitudinal trajectory of the putaminal/caudate uptake ratio in PD is depicted in Figure 3.

**Fig. 3.**
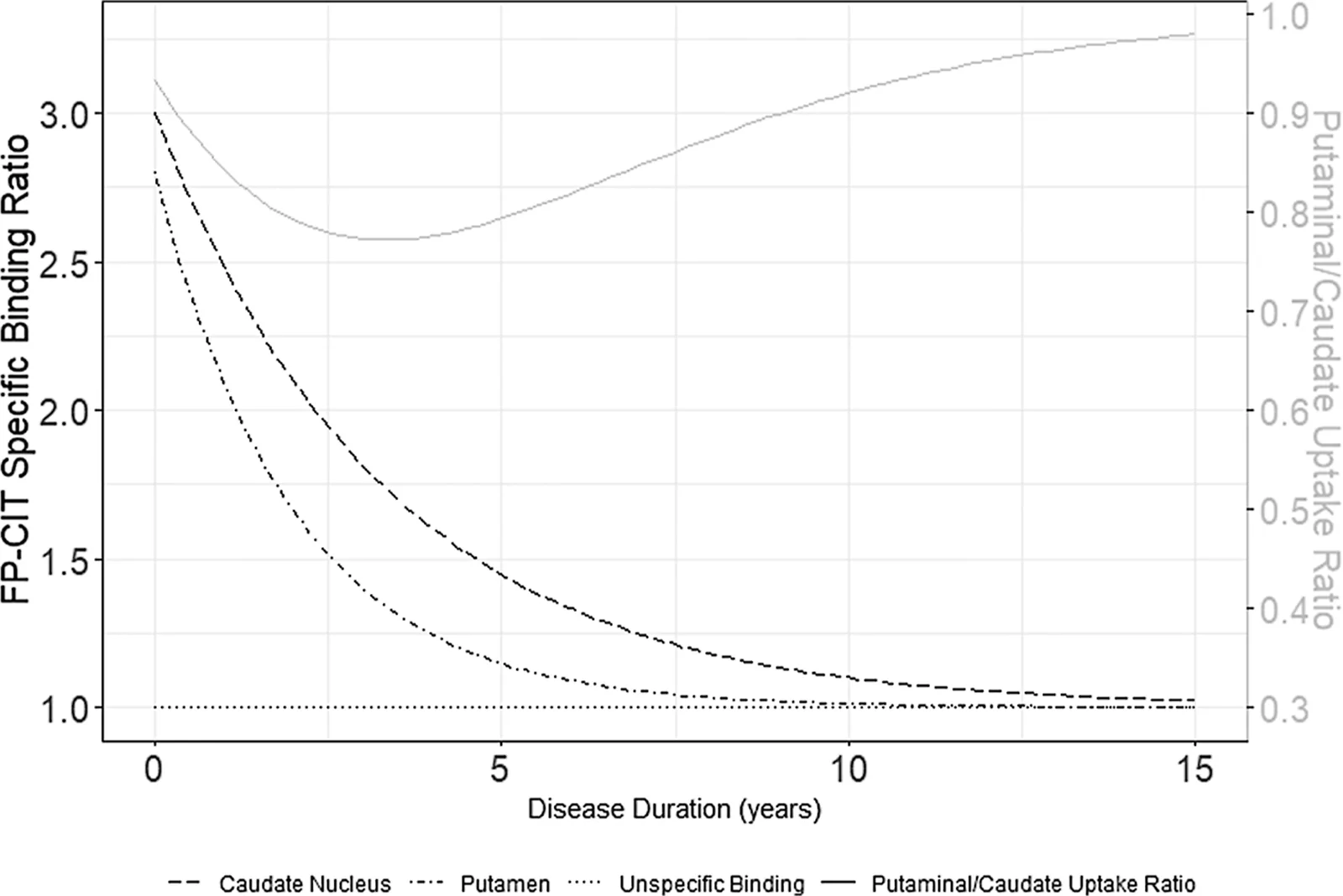
Putaminal/caudate uptake ratio during disease progression. Theoretical depiction of the specific binding ratio (SBR) of [^123^I]-FP-CIT as a function of disease duration. The left Y-axis represents the SBR, and the right Y-axis represents the putaminal/caudate uptake ratio. In healthy individuals, this ratio is about 1. In early Parkinson’s disease, it typically decreases below 1. With further disease progression, it increases again toward 1 as the caudate nucleus becomes increasingly affected.

A more pronounced early involvement of the caudate is seen in atypical Parkinsonism and in patients with PD with early cognitive changes [24, 25]. We found that more pronounced involvement of the caudate, indicated by a higher preoperative putaminal/caudate uptake ratio, was also associated with later disease stages and a higher risk of death. Consistent with this, previous studies have reported that lower striatal specific binding, particularly in the caudate nucleus, was associated with higher mortality in patients with PD and Parkinsonism not treated with DBS [26, 27]. In other studies that examined specific binding in the total striatum or putamen, but not in the caudate nucleus subregion, no association was found between SBR and mortality [28]. Together, the findings suggest that although the putamen, a key node of the motor corticobasal ganglia network, is preferentially involved in PD, greater denervation of the caudate nucleus, part of the cognitive and limbic networks, is more strongly associated with mortality [29]. Our findings underline that the putaminal/caudate uptake ratio should always be evaluated in relation to relevant clinical data and with consideration of the disease duration. This finding is thus important for the evaluation of DAT SPECT in the clinical setting.

In contrast to some studies, no significant correlations were found between striatal SBR and the clinical symptoms assessed with UPDRS motor score ON or OFF medication [3, 30, 31]. Benamer et al. [3] found that patients with unilateral symptoms had a significant difference between the striatal SBR on the two hemispheres, but with a considerable overlap. They also found that the Med OFF UPDRS correlated with the striatal SBR [3]. Rahmim et al. [32] found that UPDRS significantly correlated with the mean uptake of the more affected putamen when both healthy controls and patients with PD were included. This was, however, not significant when only patients with PD were analyzed, suggesting that the method effectively differentiates people with and without PD but not the clinical state within PD [32]. This may also be the reason why we do not find a correlation in this rather homogeneous group of patients with advanced PD.

Cognitive decline is a substantial problem for patients with PD. The Sydney Multicenter Study found that 83% of patients with a disease duration of 20 years had dementia diagnosed with neuropsychological testing [33]. Studies investigating patients with PD not treated with DBS have found that dopaminergic depletion in the caudate nucleus is associated with cognitive decline, whereas involvement of the putamen is associated with motor severity [34–36]. In our group, 30 patients developed dementia in accordance with their medical records. We did not find a higher risk of dementia in relation to preoperative striatal SBR, caudate SBR, or putaminal/caudate uptake ratio in the survival analysis. Patients were tested with the Mattis DRS before surgery, and a subset was also tested at the long-term follow-up. However, no correlation was found between Mattis DRS, either before surgery or at the long-term follow-up, and preoperative striatal SBR, putaminal/caudate uptake ratio, or caudate binding ratio.

Contrary to previous studies, we did not find a significant association between the preoperative DAT SPECT and depression [36–38]. In addition to dopamine transporters, [^123^I]FP-CIT also has some affinity for serotonin transporters; therefore, its binding is influenced by intake of drugs which modulate serotonin transporters like SSRIs and SNRIs. The use of SSRIs and SNRIs before the scan has been shown to increase [^123^I]FP-CIT binding in the basal ganglia by approximately 10% in both patients with PD and healthy controls [19, 20], potentially leading to an underestimation of dopaminergic degeneration. This effect could have concealed a potential difference between depressed and non-depressed patients with PD. However, even after correcting for the possible influence of SSRIs and SNRIs, no significant difference was found in our data.

## Limitations

This study aimed to examine how baseline DAT SPECT relates to the long-term outcome of STN-DBS in PD. However, with 55 patients, the study may still be underpowered. Dopamine transporter imaging is known to show large intra- and interindividual variability, which might necessitate a larger study population. This variability might partly explain why not all findings from previous studies could be replicated.

Furthermore, this was a descriptive study, where only 24 patients out of 55 who underwent surgery went through the detailed long-term in-person assessment, while 25 died before long-term follow-up. For nearly half of the cohort, outcome data were limited to retrospective medical record review. However, this was a description of a representative population with the causes of death as expected in an elderly population of people with PD. We did not have the possibility to assess participants who died before the long-term follow-up, but we had the description of clinical data from medical records from the department of neurology, as all individuals were seen regularly until they passed away, and changes to their clinical status were documented.

Inherently, the reported data in this study are more exploratory and descriptive than hypothesis-driven, which gives rise to type I and type II errors that are difficult to avoid. While the data are valuable to report, they are hypothesis-generating and require external validation.

Lastly, DAT SPECT is typically used early in the disease course to support the diagnosis of PD. In contrast, our study data were obtained from patients with longer disease duration, as the scans were performed before STN-DBS surgery. With disease progression, dopaminergic degeneration in the basal ganglia increases, resulting in lower DAT binding. With time, binding in the basal ganglia approaches the background level, potentially introducing artefacts. This may introduce measurement error when studying late-stage PD, impeding the utility of DAT SPECT for long-term follow-up due to the very low specific-to-nonspecific uptake.

## Conclusions

In this study, we confirmed that dopamine transporter imaging correlated with disease duration in patients with PD. Furthermore, relatively greater denervation of the caudate nucleus (reflected by a higher putaminal/caudate uptake ratio) appears to be associated with longer disease duration and higher mortality in patients with advanced PD. This indicates that the progression of the putaminal/caudate uptake ratio might follow a U-shaped rather than a linear trajectory. This is an important and relevant prognostic association that should be followed up by further prospective clinical evaluation in order to provide external validation of the finding.

Our study highlights the importance of assessing regional differences within the striatum as a more precise reflection of the disease stage and clinical status. Even the simple comparison between the caudate and putaminal uptake, performed here in a highly systematic fashion using a standardized ROI method, appeared to provide additional insight into the interpretation. A more detailed evaluation of regional striatal uptake may have clinical relevance.

This study presents a long-term follow-up of dopamine transporter imaging in patients with PD treated with STN-DBS, a highly selected group with long disease duration already at the time of imaging. Although DAT SPECT did not predict the therapeutic effect of STN-DBS, this study demonstrates the value of assessing the dopaminergic system before advanced treatment, as it may provide prognostic insight into disease progression and mortality.

## Acknowledgments

We thank Matilde Winther-Jensen for statistical support.

## Statement of Ethics

This study protocol was reviewed and approved by the National Committee on Health Research Ethics for the Capital Region of Denmark (De Videnskabsetiske Komiteer for Region Hovedstaden), approval No. H-15007736. Written informed consent was obtained for all participants who were alive at the time of the study. The study was granted exemption from the requirement of written informed consent from the individuals who had died before the study was performed by the National Committee on Health Research Ethics for the Capital Region of Denmark. The authors confirm that this research has been performed in accordance with the Declaration of Helsinki and approved by an ethics committee. We confirm that we have read the journal’s position on issues involved in ethical publication and affirm that this work is consistent with those guidelines.

## Conflict of Interest Statement

AL has received honoraria from Ipsen and AbbVie and support for course attendance from Boston Scientific. The other authors have no conflicts of interest to declare.

## Funding Sources

The Danish Parkinson’s Association has funded this project. The funder had no role in the design, data collection, data analysis, and reporting of this study.

## Author Contributions

BT: execution (research project), design (statistical analysis), execution (statistical analysis), and writing of the first draft (manuscript preparation). SJ and AC: organization (research project), execution (research project), and review and critique (manuscript preparation). SF: execution (research project), design (statistical analysis), execution (statistical analysis), and review and critique (manuscript preparation). BJ: execution (research project) and review and critique (manuscript preparation). MK: conception (research project), organization (research project), execution (research project), and review and critique (manuscript preparation). ML: conception (research project), organization (research project), execution (research project), design (statistical analysis), execution (statistical analysis), review and critique (statistical analysis), and review and critique (manuscript preparation). AL: conception (research project), organization (research project), execution (research project), design (statistical analysis), review and critique (statistical analysis), and review and critique (manuscript preparation).

## Data Availability

The data that support the findings of this study are not publicly available due to their containing information that could compromise the privacy of research participants. Data are available from the corresponding author (Annemette Løkkegaard, e-mail: annemette.loekkegaard@regionh.dk) upon reasonable request and completion of a data sharing agreement.
